# Diagnostic accuracy and prediction increment of markers of epithelial-mesenchymal transition to assess cancer cell detachment from primary tumors

**DOI:** 10.1186/s12885-017-3964-3

**Published:** 2018-01-16

**Authors:** Evan L. Busch, Prabhani Kuruppumullage Don, Haitao Chu, David B. Richardson, Temitope O. Keku, David A. Eberhard, Christy L. Avery, Robert S. Sandler

**Affiliations:** 1 Channing Division of Network Medicine, Department of Medicine, Brigham and Women’s Hospital and Harvard Medical School, 181 Longwood Avenue, 3rd Floor, Boston, MA 02115 USA; 2 Department of Epidemiology, Harvard T.H. Chan School of Public Health, Boston, MA USA; 3 Department of Epidemiology, Gillings School of Global Public Health, University of North Carolina, Chapel Hill, NC USA; 4 Department of Biostatistics and Computational Biology, Dana-Farber Cancer Institute, Boston, MA USA; 5 Department of Biostatistics, Harvard T.H. Chan School of Public Health, Boston, MA USA; 6 Department of Computer Science and Statistics, University of Rhode Island, Kingston, RI USA; 7 Division of Biostatistics, University of Minnesota School of Public Health, Minneapolis, MN USA; 8 Department of Medicine, University of North Carolina, Chapel Hill, NC USA; 9 Department of Pathology and Laboratory Medicine, University of North Carolina, Chapel Hill, NC USA

**Keywords:** Metastasis, Epithelial-msenchymal transition, Biomarker, Diagnostic accuracy, Prediction, Risk reclassification, No gold standard, Latent class, Sensitivity, Specificity

## Abstract

**Background:**

Metastases play a role in about 90% of cancer deaths. Markers of epithelial-mesenchymal transition (EMT) measured in primary tumor cancer cells might provide diagnostic information about the likelihood that cancer cells have detached from the primary tumor. Used together with established diagnostic tests of detachment—lymph node evaluation and radiologic imaging—EMT marker measurements might improve the ability of clinicians to assess the patient’s risk of metastatic disease. Translation of EMT markers to clinical use has been hampered by a lack of valid analyses of clinically-informative parameters. Here, we demonstrate a rigorous approach to estimating the sensitivity, specificity, and prediction increment of an EMT marker to assess cancer cell detachment from primary tumors.

**Methods:**

We illustrate the approach using immunohistochemical measurements of the EMT marker E-cadherin in a set of colorectal primary tumors from a population-based prospective cohort in North Carolina. Bayesian latent class analysis was used to estimate sensitivity and specificity in a setting of multiple imperfect diagnostic tests and no gold standard. Risk reclassification analysis was used to assess the extent to which addition of the marker to the panel of established diagnostic tests would improve mortality prediction. We explored how changing the latent class conditional dependence assumptions and definition of marker positivity would impact the results.

**Results:**

All diagnostic accuracy and prediction increment statistics varied with the choice of cut point to define marker positivity. When comparing different definitions of marker positivity to each other, numerous trade-offs were observed in terms of sensitivity, specificity, predictive discrimination, and prediction model calibration. We then discussed several implementation considerations and the plausibility of analytic assumptions.

**Conclusions:**

The approaches presented here can be extended to any EMT marker, to most forms of cancer, and to different kinds of EMT marker measurements, such as RNA or gene methylation data. These methods provide valid, clinically-informative assessment of whether and how to use a given EMT marker to refine tumor staging and consequent treatment decisions.

**Electronic supplementary material:**

The online version of this article (10.1186/s12885-017-3964-3) contains supplementary material, which is available to authorized users.

## Background

Metastases play a role in about 90% of cancer deaths [1], making accurate assessment of whether cancer cells have detached from the primary tumor an essential component of cancer diagnosis. Physicians use two diagnostic tests jointly to assess detachment as part of tumor staging: examination of lymph nodes near the primary tumor and radiologic imaging. While highly useful, these methods do not always successfully detect metastases. An example of this imperfect accuracy is the fact that roughly 25% of colorectal cancer (CRC) patients diagnosed with local disease later are found to have a recurrence [2]. Many of these recurrences could be due to metastases that were too small to be detected by imaging or lymph node evaluation at diagnosis. Adding a third test might substantially reduce the number of patients with false negative results across the entire panel of tests, alerting clinicians to the possible presence of metastatic disease that might otherwise have gone undetected. This could lead to more appropriate adjuvant chemotherapy decisions for patients who stand to benefit from it.

Given that roughly 80% of cancer originates in epithelial cells [1], markers of epithelial-mesenchymal transition (EMT), a mechanism of metastasis, might be able to serve as a third test of detachment [3]. The mechanism consists of increased cellular expression of EMT inducers leading to temporarily decreased expression of epithelial markers and increased expression of mesenchymal markers [4]. These molecular changes promote detachment and cellular motility. An EMT marker can be any gene or molecule—inducer, epithelial marker, or mesenchymal marker—for which the cellular expression level would be expected to temporarily change as part of the process of EMT.

Measurements of EMT marker expression in primary tumor cancer cells at diagnosis could suggest whether the tumor contained a substantial number of cells that were capable of detaching, thereby informing assessment of the risk that cancer cells had already detached [3]. Used in this way, they could refine the accuracy of tumor staging. Such EMT measurements have often, though not always, been associated with patient outcomes [3, 5–10]. However, to our knowledge, no study has evaluated the ability of an EMT marker to assess cancer cell detachment from the primary tumor in terms of several parameters that are more relevant than measures of association to deciding whether and how to use the marker clinically to refine tumor staging: sensitivity and specificity (collectively “diagnostic accuracy”), as well as improvement of patient outcomes prediction when adding the marker to standard tests compared to prediction based only on standard tests (i.e. “prediction increment”).

Estimating diagnostic accuracy and prediction increment is complicated by the fact that imaging and lymph node evaluation, considered singly or jointly, do not predict individual patient outcomes perfectly, nor do they have 100% sensitivity and 100% specificity to assess detachment. Latent class analysis provides a way to estimate the sensitivity and specificity of a diagnostic test in settings with multiple imperfect tests but no gold standard [11–13]. Risk reclassification analysis is an approach to statistical prediction that can evaluate the prediction increment of a new predictor by comparing classification of individuals as high risk or low risk for an outcome of interest based on established predictors to classification based on established predictors plus the new predictor [14–16].

The purpose of this paper is to demonstrate, for any EMT marker measured in primary tumor cancer cells from virtually any kind of cancer, how to use latent class analysis to estimate the sensitivity and specificity of the marker to assess detachment, and how to use risk reclassification to evaluate an outcomes prediction increment of the marker. To illustrate, we provide a worked example using immunohistochemical (IHC) measurements of the EMT epithelial marker E-cadherin measured in a cohort of CRC tumors, then discuss analytic assumptions and recommendations for the design of future studies. These statistical approaches can be extended to forms of EMT marker data other than IHC measurements, such as RNA or gene methylation data. Although our focus is on markers of EMT, the methods presented here can be applied to any candidate marker of detachment that could be measured in primary tumor cancer cells, regardless of whether the marker is implicated in the mechanism of EMT.

## Methods

The study population [6, 17–19], laboratory work [6], IHC digital image analysis [6], and image scoring methods [6] have been described previously. Representative staining images are in Additional file 2 (Figure S1). Here, we present only those details needed to understand the approach to estimating diagnostic accuracy and prediction increment.

### Study population

Subjects were 188 CRC patients enrolled in the North Carolina site of the Cancer Care Outcomes Research and Surveillance Consortium (CanCORS) for whom E-cadherin was measured in primary tumor specimens [6]. CanCORS was a population-based, case-only, multi-site prospective cohort study of colorectal and lung cancer that enrolled subjects during 2003–06 [17]. North Carolina enrolled only CRC patients and was the only site to collect tissue specimens. Survey and medical records data were collected, including physician-diagnosed TNM tumor stage [19]. Tissue microarrays were constructed for primary tumor and tumor-adjacent specimens [6, 18]. The present analysis used E-cadherin measurements from tumor tissue only. The protocol was approved by the Institutional Review Board of the University of North Carolina at Chapel Hill, and all subjects provided written informed consent.

### E-cadherin measurements

Protein expression of E-cadherin was measured in epithelial cancer cells in primary tumor tissue cores, generally with 3 cores per subject. Regions of each core image other than epithelial cells were excluded from analysis.

For an individual cell, E-cadherin measurement included plasma membrane and cytoplasmic staining, but excluded nuclear staining. For included cells within a core, staining intensity was measured for each cell, then averaged across all cells to produce a continuous average intensity score for that core on a scale of 0–3. To collapse scores from multiple cores for the same person into a summary score, the core average intensities were combined as a weighted average, weighted by the amount of core area analyzed.

### Diagnostic tests of detachment

Except when E-cadherin was included in a prediction model as a continuous variable, each test was coded as dichotomous test-positive versus test-negative. To make the interpretation of test results consistent across all tests, each test was coded so that a positive result meant evidence supporting detachment and a negative result meant no evidence of detachment.

Test results for lymph node evaluation and imaging were not available in CanCORS. These two tests were handled differently for estimation of diagnostic accuracy than for estimation of prediction increment (see Discussion for rationale). For models of diagnostic accuracy, information from prior literature about lymph node evaluation and imaging was used to develop Bayesian priors for these tests. See the Statistical Analysis section below and Additional file 1 for further details. When estimating various statistical measures of prediction increment, results for lymph node evaluation and imaging were imputed based on each subject’s TNM tumor stage as follows:

Local disease (Stage I or II) was assigned as lymph node-negative and imaging-negative; regional disease (Stage III) as lymph node-positive and imaging-negative; and distant disease (Stage IV) as imaging-positive, with lymph node status assigned using the N-stage component of overall stage when available. Among 23 subjects diagnosed with distant disease, 3 who were N0 were assigned as lymph node-negative, 11 who were N1 or N2 were assigned as lymph node-positive, and 9 with unknown N-stage were assigned as lymph node-positive.

Lymph node-positive status meant that cancer cells had been observed in the regional lymph nodes, while lymph node-negative meant that no cancer cells had been observed in the nodes. Imaging-positive meant that evidence of cancer cells away from the primary tumor had been observed in an imaging study (e.g. MRI, CT), while imaging-negative meant that all scans performed on the patient had been interpreted as not showing evidence of cancer cells away from the primary tumor.

The coding of the EMT diagnostic test was based on the biological role of E-cadherin in EMT. E-cadherin is an epithelial cell transmembrane protein that serves as a critical adhesion molecule between adjacent epithelial cells, helping to anchor the cells in the epithelial layer [1, 4]. Loss of plasma membrane E-cadherin expression is thought to be a key step in a cancer cell detaching from the primary tumor. Consequently, E-cadherin membrane expression would be expected to be low in an epithelial cell undergoing EMT and high in an epithelial cell not undergoing EMT. For a given cut point dichotomizing continuous E-cadherin expression into high expression (at or above the cut point) and low expression (below the cut point) groups, low E-cadherin was considered EMT-positive and high E-cadherin was considered EMT-negative.

For an EMT inducer or mesenchymal marker—either of which increases in expression during EMT—high expression would be coded as EMT-positive and low expression as EMT-negative, but the present example uses only an epithelial marker.

### Diagnostic accuracy conceptual model

**Fig. 1:**
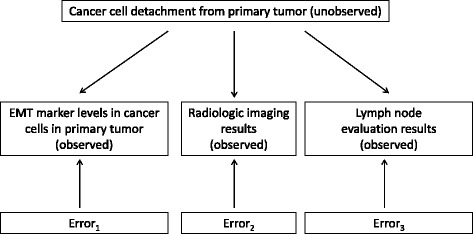
Latent class model of cancer cell detachment from primary tumors and diagnostic tests of detachment (EMT, epithelial-mesenchymal transition)

To estimate EMT marker sensitivity and specificity, we conceived the relationship between cancer cell detachment and the diagnostic tests that assess it in terms of the latent class framework (Fig. 1) [12]. Detachment was the latent variable, meaning the variable that was of prime interest but was not observed directly. We thought of the latent variable as binary, or in other words, as having two latent classes: tumors from which substantial numbers of cancer cells had, or had not, detached. For present purposes, it was not necessary to define “substantial numbers” quantitatively. We merely needed to group tumors as high risk or low risk for metastatic disease based on observed patterns of diagnostic test results.

The latent variable was measured indirectly by the three diagnostic tests: E-cadherin measurements in primary tumor cancer cells, lymph node evaluation, and imaging. The observed data for each diagnostic test were regarded as being generated jointly by the extent of detachment and a random error term specific for that test.

### Statistical analysis

Estimation of E-cadherin sensitivity and specificity to assess detachment was conducted using Bayesian latent class analysis following the framework of Zhang et al. [20]. Based on our previous finding that dichotomous E-cadherin defined by cut points of 0.52, 0.60, and 0.85 were each associated with time to all-cause mortality in this dataset [6], we estimated the diagnostic accuracy of each of these dichotomous E-cadherin variables.

Let *Y*_*ij*_ be the classification result of the *j*^*th*^ of three tests (EMT, lymph node evaluation, and imaging) for individual *i (i = 1, …, n*), the latent variable *D*_*i*_ denote the true disease status, and π_i_ denote the disease probability of the *i*^*th*^ subject. The correlation in disease misclassification is accommodated by a latent continuous variable *Z*_*i*_ ~ *N*(*0, 1*). The positive result for the *j*^*th*^ assessment is assumed to depend on both the latent true disease status *D*_*i*_ of the *i*^*th*^ subject and the Gaussian latent variable *Z*_*i*_ through a generalized linear mixed regression model, such as a probit model [20],

$$ P\left({Y}_{ij}=1|{D}_i={d}_i,{Z}_i={z}_i\right)=\Phi \left({a}_{d_ij}+{c}_{d_ij}{z}_i\right) $$

where *d*_*i*_ = 0,1. Here, the latent Gaussian random variable *Z*_*i*_ is assumed to be independent of the latent disease status *D*_*i*_. Based on Zhang et al. [20], the likelihood for latent class estimation in our 3-test setting is:

$$ L\left(\theta \right)=\prod \limits_{i=1}^n\left\{{\pi}_i\underset{-\infty }{\overset{\infty }{\int }}\prod \limits_{j=1}^3\left\{\Phi {\left({a}_{1j}+{c}_{1j}{z}_i\right)}^{y_{ij}}\left[1-\Phi {\left({a}_{1j}+{c}_{1j}{z}_i\right)}^{1-{y}_{ij}}\right]\right\}d\Phi \left({z}_i\right)+\left(1-{\pi}_i\right)\underset{-\infty }{\overset{\infty }{\int }}\prod \limits_{j=1}^3\left\{\Phi {\left({a}_{0j}+{c}_{0j}{z}_i\right)}^{y_{ij}}\left[1-\Phi {\left({a}_{0j}+{c}_{0j}{z}_i\right)}^{1-{y}_{ij}}\right]\right\}d\Phi \left({z}_i\right)\right\} $$

For Bayesian estimation of diagnostic accuracy, lymph node evaluation and radiologic imaging were each assigned a prior distribution of sensitivity 60–70% and specificity 95–99%. Additional file 1 provides the rationale for assigning this prior to each of these two tests, as well as sample WinBUGS code for the latent class analysis. E-cadherin was assigned an uninformative prior. Bayesian analyses were performed using Markov Chain Monte Carlo methods with a burn-in period of 5000 iterations followed by chains of 50,000 iterations, with initial values generated by a fixed seed.

In the latent class framework, when errors for different diagnostic tests are not correlated with each other, those tests are said to be conditionally independent, that is, independent within each latent class [11, 12]. However, it is possible that errors for different tests are correlated with each other, meaning those tests would not be independent after conditioning on latent class. Per Zhang et al., for Bayesian estimates of diagnostic accuracy, we assessed the sensitivity of the results to different assumptions about conditional dependence among the tests [20].

Analysis of the outcomes prediction increment of E-cadherin used all-cause mortality as the outcome and consisted of a combination of receiver operating characteristic [ROC] curves, risk stratification tables and reclassification statistics [15, 16]. The predicted probability of death was estimated for each subject using Cox proportional hazards models of time from diagnosis to all-cause mortality, censored at 5 years after diagnosis. The base prediction model had independent variables of the established diagnostic tests (lymph node evaluation and radiologic imaging). Predicted probabilities from the base model were compared to predicted probabilities from a model with independent variables of the established tests and E-cadherin. Across multiple runs, the model with E-cadherin was implemented with different forms of the E-cadherin variable: continuous or dichotomized at 0.52, 0.60, or 0.85.

Prediction models were evaluated using four measures of discrimination—area under the ROC curve for censored outcomes (c-index; range: 0% to 100%) [15], Integrated Discrimination Improvement (IDI; range: −100% to 100%) [21], event Net Reclassification Index (event NRI; range: −100% to 100%) [22], and non-event NRI (range: −100% to 100%) [22]—as well as a goodness-of-fit test of calibration (reclassification calibration statistic) [15]. For model discrimination, larger values meant better discrimination according to all four measures. Statistically significant reclassification calibration statistic *p*-values at alpha = 0.05 indicated poor calibration.

To construct risk stratification tables as well as calculate NRI and reclassification calibration statistics, mortality risk category cut points of 20%, 30%, and 40% were chosen based on the observed distribution in the base prediction model of individual-level predicted probabilities of death. Confidence intervals and p-values for c-indices and reclassification statistics were obtained using 1000 bootstrap samples.

Bayesian latent class analysis was performed using WinBUGS 1.4.3 (Medical Research Council Biostatistics Unit, United Kingdom). All other analyses were performed using SAS 9.4 (SAS Institute, Cary, NC). C-indices and risk reclassification statistics were estimated using SAS macros available via the Brigham and Women’s Hospital Division of Preventive Medicine Risk Prediction Modeling website [23].

## Results

Subjects were mainly non-Hispanic whites, about evenly divided by sex, and had a mean age close to the United States national average age at diagnosis for colorectal cancer patients (Table 1) [24]. Of 188 subjects, 89 (47%) were diagnosed with regional or distant disease, which is similar to the approximately 56% of colorectal cancer cases in the United States diagnosed with regional or distant disease [24]. 62 subjects died within 5 years of diagnosis (Additional file 2: Tables S1-S4).

**Table 1:** Subject characteristics (*n* = 188)

	Mean	SD
Age (years)	67	13
	N	%
Sex		
Male	89	47
Female	99	53
Race		
Non-Hispanic White	149	79
Hispanic or non-White	39	21
Tumor Stage		
Local (I or II)	99	53
Regional (III)	66	35
Distant (IV)	23	12
Lymph Node Diagnostic Status^a^		
Positive	86	46
Negative	102	54
Radiologic Imaging Diagnostic Status^a^		
Positive	23	12
Negative	165	88
EMT Diagnostic Status, Cut Point = 0.52^b^		
Positive	11	6
Negative	177	94
EMT Diagnostic Status, Cut Point = 0.60^b^		
Positive	28	15
Negative	160	85
EMT Diagnostic Status, Cut Point = 0.85^b^		
Positive	108	57
Negative	80	43

^a^Inferred from tumor stage. See Methods section for assignment rules

^b^Based on E-cadherin expression in primary tumor cancer cells measured as a weighted average of tumor cores on a continuous average intensity scale of 0–3. Low E-cadherin expression (below the cut point) is evidence of EMT (EMT-positive)

*EMT* Epithelial-mesenchymal transition

Bayesian latent class estimates of diagnostic accuracy are presented in Table 2. Across different E-cadherin cut points and assumptions about conditional dependence of diagnostic tests, the sensitivity of E-cadherin ranged from 46% to 57%. The specificity of E-cadherin varied more widely, ranging from 14% to 49%.

**Table 2:** Bayesian latent class estimates of sensitivity and specificity of E-cadherin measurements in colorectal primary tumor cancer cells to assess cancer cell detachment from primary tumor (*n* = 188)

	Fully		Fully		Partial Dependent Models											
	Independent		Dependent		PDM 1		PDM 2		PDM 3		PDM 4		PDM 5		PDM 6	
Constraints Set to 0	All		None		Se(Ecad)		Se(LN)		Se(RI)		Se(Ecad)		Se(Ecad)		Se(LN)	
					Sp(Ecad)		Sp(LN)		Sp(RI)		Sp(Ecad)		Sp(Ecad)		Sp(LN)	
											Se(LN)		Se(RI)		Se(RI)	
											Sp(LN)		Sp(RI)		Sp(RI)	
E-cadherin Variable	Se	Sp	Se	Sp	Se	Sp	Se	Sp	Se	Sp	Se	Sp	Se	Sp	Se	Sp
Dichotomized at 0.52	47	28	54	44	52	49	52	42	53	44	47	28	52	48	52	43
Dichotomized at 0.60	49	22	57	43	53	40	53	41	53	43	49	22	53	40	52	42
Dichotomized at 0.85	46	14	53	43	52	41	52	42	53	43	46	14	52	41	52	41

Cellular membrane E-cadherin expression measured as protein in primary tumor cancer cells on a continuous average intensity scale (0–3), then dichotomized at the indicated cut point (coded EMT positive versus EMT negative). LN and RI were each coded as dichotomous positive versus negative. LN and RI each assigned the same prior of Se 60–70% and Sp 95–99%. For all dichotomous test variables, positive results mean evidence supporting detachment of cancer cells from the primary tumor and negative results mean no evidence of detachment. All Se and Sp estimates are reported as percentages

*Ecad* E-cadherin, *LN* lymph node evaluation, *PDM* partial dependent model, *RI* radiologic imaging, *Se* sensitivity, *Sp* specificity

When lymph node evaluation and radiologic imaging were the only predictors of all-cause mortality, the distribution of individual predicted probabilities ranged from 22% to 69%, with most subjects having the minimum probability of 22% (Table 3). Addition of E-cadherin measurements to the panel of predictors consistently lowered the minimum, and raised the maximum, of the range of predicted mortality probabilities. Including E-cadherin in the panel also increased the variation observed within the range of predicted risks, with a smaller proportion of subjects having the minimum predicted probability for the model compared to the model without E-cadherin. However, the extent of the increase in range and variation of predicted probabilities due to addition of E-cadherin to the model depended on the form of the E-cadherin variable. Greater variation was introduced into the set of predicted probabilities when adding E-cadherin that was continuous or dichotomized at 0.85 compared to dichotomization at 0.52 or 0.60. Graphical comparisons of predicted probabilities based only on established diagnostic tests to predicted probabilities based on established diagnostic tests and E-cadherin are in Additional file 2 (Figures S2-S5).

**Table 3:** Distributions of predicted probabilities for individuals of all-cause mortality within 5 years of diagnosis among colorectal cancer patients for prediction models with and without primary tumor E-cadherin measurements (*n* = 188)

		Predicted Mortality Probability as Percentage						
Model	Range	10th percentile	25th percentile	50th percentile	75th percentile	90th percentile	Mean	SD
Lymph Node Evaluation + Radiologic Imaging (Base Model)	22–69	22	22	22	38	69	33	14
Base Model + Continuous E-cadherin	9–87	15	21	29	41	60	33	17
Base Model + E-cadherin Dichotomized at 0.52	20–79	20	20	35	35	69	33	17
Base Model + E-cadherin Dichotomized at 0.60	19–93	19	19	33	40	67	33	17
Base Model + E-cadherin Dichotomized at 0.85	15–83	15	25	28	45	58	33	17

Base Model includes standard diagnostic tests of lymph node evaluation and radiologic imaging (each coded as dichotomous positive versus negative). Each of the other models includes standard diagnostic tests and cellular membrane E-cadherin expression measured by immunohistochemistry in primary tumor cancer cells on a continuous average intensity scale (0–3), then modeled as continuous or dichotomized at the indicated cut point (if dichotomized, then coded as dichotomous EMT positive versus EMT negative). For all dichotomous predictors, a positive result means evidence supporting detachment of cancer cells from the primary tumor and a negative result means no evidence of detachment. Each model is a Cox proportional hazards models of time from cancer diagnosis to all-cause mortality, censored at 5 years after diagnosis. 62 subjects died within 5 years of diagnosis

Table 4 presents c-indices and risk reclassification statistics; corresponding risk stratification tables (Tables S1-S4) are in Additional file 2. The c-index for a model of lymph node evaluation and imaging was 45% (95% CI 36%, 53%) (Table 4). Addition of E-cadherin to the model increased the c-index, though the magnitude of the change depended on the form of the E-cadherin variable and the confidence intervals for most of the estimates with E-cadherin overlapped with the confidence interval for the estimate without E-cadherin. Continuous E-cadherin produced the largest increase, with more modest increases for different dichotomous E-cadherin variables.

**Table 4:** Prediction of all-cause mortality after adding continuous or dichotomous E-cadherin measurements to standard diagnostic tests of cancer cell detachment from colorectal primary tumors (*n* = 188)

	E-cadherin Variable Added to Standard Tests			
		Dichotomous E-cadherin Cut Point		
	Continuous	0.52	0.60	0.85
C-Index, % (95% CI)^a^	66 (58, 72)	51 (41, 59)	54 (45, 62)	56 (48, 63)
Reclassification Metric^b^				
Number (%) moved to higher risk category	47 (25)	11 (6)	27 (14)	41 (22)
Number (%) moved to lower risk category	55 (29)	93 (49)	83 (44)	70 (37)
Total number (%) reclassified	102 (54)	104 (55)	110 (59)	111 (59)
Reclassification Calibration Statistic P-value	0.1	0.1	0.1	0.2
Event Net Reclassification Index, % (95% CI)	14 (−11, 30)	−22 (−38, −7)	−7 (−23, 10)	3 (−15, 21)
Non-Event Net Reclassification Index, % (95% CI)	13 (3, 35)	54 (44, 63)	41 (29, 52)	24 (12, 37)
Integrated Discrimination Improvement, % (95% CI)	3.4 (1.9, 5.6)	4.3 (2.2, 6.8)	3.4 (1.8, 5.3)	3.7 (1.7, 5.9)

E-cadherin measured on a continuous average intensity scale of 0–3, then modeled as either continuous or dichotomized (EMT positive versus EMT negative) at a given cut point. For dichotomous E-cadherin, EMT positive status was expression below the cut point while EMT negative status was expression at or above the cut point. 62 subjects died within 5 years of diagnosis

^a^Each c-index value in the table is for a Cox model estimating 5-year risk of all-cause mortality based on standard diagnostic tests of cancer cell detachment (lymph node evaluation and radiologic imaging) plus the respective continuous or dichotomous E-cadherin variable. C-index for a model of standard diagnostic tests only was 45% (95% CI 36%, 53%)

^b^Reclassification metrics compare a Cox model estimating 5-year risk of all-cause mortality based on standard diagnostic tests of cancer cell detachment (lymph node evaluation and radiologic imaging) to a Cox model based on standard diagnostic tests plus continuous or dichotomous E-cadherin status defined by a given cut point. Mortality risk categories were 0 – 20%, 20 – 30%, 30 – 40%, and > 40%

All prediction models with E-cadherin were well-calibrated (Table 4). Addition of E-cadherin to the panel of predictors increased prediction model discrimination according to the IDI, which had a similar magnitude across all forms of the E-cadherin variable that we examined. Addition of E-cadherin to the model presented different trade-offs between the event NRI and non-event NRI that depended on the form of the E-cadherin variable.

## Discussion

We demonstrated how to obtain valid, clinically-informative estimates of the sensitivity, specificity, and mortality prediction increment of an EMT marker measured in primary tumor cancer cells to assess cancer cell detachment from the primary tumor. The approaches can be applied to any EMT marker, for most forms of cancer, and to different types of EMT marker measurements, such as protein, RNA, or gene methylation data. The approach to estimating prediction increment can also be applied to outcomes other than all-cause mortality, such as cancer-specific mortality or metastasis-free survival. In our worked example, we observed that the choice of cut point to define marker positivity influenced all of the statistics we examined and that numerous trade-offs existed between different cut points. The statistical approaches and consideration of many different cut points to define marker positivity can help to evaluate whether and how an EMT marker should be used clinically to assess detachment and refine tumor staging for the form of cancer under study. By improving the accuracy of tumor staging, successful addition of an EMT marker to the established diagnostic tests of lymph node evaluation and radiologic imaging could lead to more appropriate treatment decisions.

Proper interpretation of the results requires that they be viewed in the context of several assumptions and analytic considerations. Lymph node evaluation and radiologic imaging each provide diagnostic information on two related questions: have cancer cells detached from the primary tumor, and if so, where are they? EMT marker measurements in primary tumor cancer cells can inform the first question but not the second. These measurements are cross-sectional in time and only taken in a sample of the primary tumor cancer cells after resection. Consequently, diagnostic use of EMT markers assumes that the observed EMT expression provides an informative representation of whether cancer cells were likely to have detached from the primary tumor as a whole at some point during the history of the tumor from initiation to surgery. This assumption would be difficult to evaluate, but it is probably reasonable to the extent that the behavior of the tumor is stable in the weeks or months immediately before surgery.

Another assumption is conditional independence of diagnostic test errors [12]. Whether errors for different diagnostic tests are correlated with each other cannot be evaluated in a frequentist latent class analysis when there are fewer than four tests, but can be evaluated in a Bayesian analysis due to the use of priors [20]. While we had three tests, our Bayesian estimates suggested that the estimate of E-cadherin sensitivity did not vary much by dependence assumptions, but the estimate of specificity did vary by dependence assumptions.

In general, each test is measured in a different part of the body, using different technology, and evaluated by different personnel. Lymph nodes are taken from near the primary tumor and examined under a microscope by a pathologist. While imaging is sometimes performed on the regional lymph nodes, it is usually applied to parts of the body distant from the primary tumor, uses imaging machines such as MRI or CT, and is evaluated by a radiologist. As envisioned here, clinical measurement of EMT markers would be performed in the primary tumor and could be automated to scoring by computer. Based on these qualitative considerations and our findings, the three diagnostic tests probably do not strictly meet the conditional independence assumption, but could be reasonably close to satisfying it.

Our estimation of the prediction increment of E-cadherin in CRC must be interpreted as assessing the extent to which the marker improves the ability of tumor staging to predict all-cause mortality. It does not assess the extent to which E-cadherin measurements improve the ability of the total set of clinical CRC predictors to predict all-cause mortality, as additional predictors besides stage could include age, tumor grade, and comorbid conditions, among other variables. While the prediction increment of an EMT marker is of interest in both cases—in relation to stage and to the full panel of predictors—adequate data were not available in CanCORS to evaluate the prediction increment for the full panel. Nevertheless, the major clinical role of EMT markers would be to refine tumor staging.

Our approach to evaluating an EMT marker had several notable strengths. First, the latent class framework provided a realistic description of the relationship between cancer cell detachment from primary tumors, which is never directly observed in the clinic, and the diagnostic tests that assess it. More importantly, the framework permitted estimation of the diagnostic accuracy of an EMT marker in a setting with multiple imperfect tests but no gold standard. Cross-tabulation calculations of sensitivity and specificity based on a 2 × 2 table assume that the new test is being compared to an absolute gold standard having both 100% sensitivity and 100% specificity [25]. While lymph node evaluation and radiologic imaging perform well to assess detachment, especially when used together, their excellent joint performance does not constitute an absolute gold standard. By avoiding this assumption, latent class analysis gave more valid estimates than would cross-tabulation calculations.

Also advantageous was our use of Bayesian estimation of diagnostic accuracy. With fewer than four diagnostic tests, statistical theory shows that frequentist estimates of a fully dependent model are not identifiable, although partially dependent models can be assessed [20]. Bayesian estimation overcomes this limitation for a fully dependent model by the use of priors, which can also enhance the analysis because the informative priors for lymph node evaluation and imaging can be based on information from much larger datasets than our own.

A further strength of our approach was the use of risk reclassification analysis to estimate the prediction increment of an EMT marker. Unlike measures of association [26] or ROC curves [27], reclassification is a prediction method that focuses on individual-level assignment of subjects with different values for predictors into clinically-relevant risk categories [16]. We showed how the distribution of individual predicted probabilities of dying within 5 years of CRC diagnosis displayed greater variation when going from prediction based only on lymph node evaluation and imaging to prediction based on lymph node evaluation, imaging, and E-cadherin (Table 3).

Finally, our use of digital image analysis to obtain continuous marker expression data allowed us the deepest possible assessment of whether and how E-cadherin should be used clinically to improve tumor staging. Besides comparing the impact of modeling continuous versus dichotomous marker expression in the reclassification analysis, we compared different dichotomous marker expression variables to each other, with each defined by a different cut point. Previously, we noted several trade-offs between E-cadherin cut points to define marker positivity: the proportion of subjects defined as high expression as well as the magnitude and precision of the association with all-cause mortality [6].

The present analysis showed that the trade-offs between cut points are even more extensive than our previous report demonstrated. Varying the cut point changed the range and distribution of individual predicted probabilities (Table 3), the improvement across different measures of prediction model discrimination (c-index, event NRI, non-event NRI, IDI) (Table 4), and how well calibrated the prediction model was (Table 4). Curiously, as the cut point to dichotomize continuous E-cadherin was raised to increase the proportion of subjects considered EMT-positive, sensitivity did not necessarily increase and specificity decrease accordingly (Table 2). While this may have been due to idiosyncrasies of our small dataset, in general one would expect to find such sensitivity-specificity trade-offs as the marker cut point changes.

The dataset used for the example analysis had several limitations. The small sample size, unavailability of outcomes other than time to all-cause mortality (e.g. cancer-specific mortality, recurrence, response to therapy), and lack of consistent sampling of similar portions across tumors—such as sampling the invasive front of each tumor—have been noted before [6]. When running prediction models to estimate risk reclassification statistics, not having test results available for lymph node evaluation and imaging forced us to infer them from tumor stage. While our rules for inferring results of established diagnostic tests based on tumor stage were probably reasonably accurate, we had no way to verify this and doubtless our imputations do not exactly reflect what was observed clinically. Since most datasets that could be used for similar analyses probably include tumor stage but not test results, our assignment rules could be useful for carrying out risk reclassification analyses in other datasets.

The lack of systematic sampling of certain portions of each tumor deserves special attention. It is possible that the diagnostically important information is EMT marker expression at the invasive front rather than other parts of the tumor or average expression throughout the tumor. Ideally, for each tumor, one would want to sample both invasive front and tumor center, then run separate analyses for expression data from the two locations. However, in CanCORS, the tumors were sampled randomly, so that the available tumor specimens were an unknown mixture of invasive front, tumor center, and other parts of the tumors. This meant that our results could be different from what would be observed using expression data exclusively from the invasive front of each tumor.

A final limitation that was not specific to this dataset was the availability of just three diagnostic tests of detachment, since besides the candidate test (EMT markers measured in primary tumor cancer cells) there are only two tests used in current practice. This prevented us from formally evaluating correlations of errors among the tests and meant that frequentist estimates of diagnostic accuracy were not identifiable for a fully-dependent model. Note that measuring multiple EMT markers would not overcome this limitation because expression of EMT markers in a given set of tumor samples would be expected to be correlated.

In our CRC example, E-cadherin measurements had a modest sensitivity of about 50% (Table 2) that, when used together with lymph node evaluation and radiologic imaging, could provide an additional opportunity to avoid false negative test results across the entire panel of tests, thereby decreasing the number of false diagnoses of local disease. The major potential drawback would be the apparently high number of false positive results that E-cadherin might introduce, given its low estimated specificity. Whether E-cadherin also improved the ability of stage to predict patient mortality was unclear (Table 4). Across different forms of the E-cadherin variable, models with E-cadherin were well calibrated and had a positive IDI. However, there were clear trade-offs between the event NRI and non-event NRI, especially when looking across multiple forms of the E-cadherin variable. Given that dichotomous E-cadherin is more easily interpretable in a clinical setting than continuous E-cadherin, the small or even negative event NRI values for the dichotomous E-cadherin variables suggest that the marker might not improve identification of patients at the greatest risk of dying.

As noted earlier, test results for lymph node evaluation and imaging were not available in CanCORS. We incorporated information about these tests into estimation of both EMT marker diagnostic accuracy and prediction increment, but the manner of doing so was different in each case. For diagnostic accuracy, we accounted for lymph node evaluation and imaging using informative Bayesian priors that were developed using external information. For estimation of prediction increment statistics, we imputed test results based on each person’s TNM stage. Each approach seemed appropriate for the statistics for which it was used. Sensitivity and specificity are calculated using group-level data, making the use of external population data about the diagnostic accuracy of lymph node evaluation and imaging a valid method, especially if the external data is based on a much larger sample. However, the usefulness of risk reclassification statistics is closely tied to the change in an individual’s predicted probability of the outcome when classified with and without the new predictor. For prediction increment, it seemed best to use values for lymph node evaluation and imaging test results that were specific for that individual, even if these had to be inferred from other information such as tumor stage.

Given the use of IHC data in this analysis, we note the decision to restrict digital image analysis to the epithelial cells in each image. Non-epithelial cells could potentially be cancer cells that have undergone a complete EMT. However, if a cell has undergone a complete EMT, it might not be clear whether it is a cancer cell at all, and might have already detached from the primary tumor. The potential diagnostic test considered in this manuscript is the measurement of EMT markers in cancer cells that are still attached to the primary tumor, but which might or might not be in the process of detaching at the time of tumor resection. The idea is to get an assessment of risk of metastatic disease based exclusively on measurements in the primary tumor, regardless of whether any detached cancer cells are observed. This is what makes the test a genuine third test of risk of metastatic disease, independent of radiologic imaging and lymph node evaluation. Given the aim of the proposed diagnostic test, it seemed reasonable to restrict analysis to those cells retaining at least some epithelial morphology.

For design of future analyses of EMT marker diagnostic accuracy and prediction increment, Table 5 presents several implementation recommendations specific for the topic, which expand upon our earlier work [3]. General principles of biomarker evaluation, such as the importance of external validation, are omitted but remain relevant. When interpreting results for the prediction increment of an EMT marker, we recommend emphasizing reclassification statistics such as event NRI, non-event NRI, and the reclassification calibration statistic over measures based on ROC curves (such as the c-index) because the reclassification framework is more readily interpretable in terms of clinical settings than ROC curves [16]. EMT marker expression should be measured as continuous data whenever possible, such as by using digital image analysis of IHC staining. After obtaining continuous data, and given that dichotomous EMT marker status corresponds to a clinical decision more readily than continuous expression, we suggest evaluating the diagnostic accuracy and prediction increment of every possible cut point that the data permit to dichotomize EMT marker expression. An example of this kind of exhaustive cut point analysis is provided in Tables 2, 3, and 4 of a paper by Busch et al. [28], though in the present context, the consideration of many cut points would be applied to estimation of diagnostic accuracy and various measures of prediction increment.

**Table 5:** Study design recommendations for future analyses of EMT marker diagnostic accuracy and prediction increment

1. Use Bayesian estimation of diagnostic accuracy latent class models, as the use of priors avoids the identifiability problem of three diagnostic tests (lymph node evaluation, radiologic imaging, and EMT marker expression in primary tumor cancer cells)
2. Estimate EMT marker prediction increment for both tumor stage and full panel of predictors of a given outcome. Models for stage would consist of tumor stage as the base predictor (either overall TNM stage or component T-stage, N-stage, and M-stage) to which EMT marker expression is added as a new predictor. Models for the full panel could include, in addition to tumor stage, other predictors of the outcome—such as age, tumor grade, or tumor subtype—as appropriate.
3. Estimate EMT marker prediction increment using a variety of outcomes, e.g. time to all-cause mortality, time to cancer-specific mortality, time to recurrence or metastasis-free survival, response to therapy.
4. When interpreting prediction increment results, give greater emphasis to risk reclassification statistics than ROC curves or measures of association. For reclassification, examine the event NRI and non-event NRI separately. Do not rely exclusively on the overall NRI that sums the event NRI and non-event NRI together.
5. Sample primary tumors systematically so that EMT marker expression can be measured in, for example, both the invasive front and the center of every tumor. Present portion-specific estimates in manuscripts, for example, diagnostic accuracy and prediction increment for EMT marker expression at the invasive front, and separately, diagnostic accuracy and prediction increment for EMT marker expression at the center of the tumor.
6. Measure multiple EMT markers in the primary tumors, preferably at least one for which expression goes down during EMT (epithelial markers) and at least one for which expression increases during EMT (mesenchymal markers and/or EMT inducers).
7. Measure multiple forms of EMT marker data (e.g. protein expression, RNA expression, gene methylation) in the same set of tumors to evaluate which type of data has the best diagnostic accuracy or prediction increment.
8. Measure EMT marker expression as continuous data whenever possible. Starting from continuous EMT marker expression data, create as many dichotomous EMT marker status variables defined by different cut points as the data permit, then evaluate the diagnostic accuracy and prediction increment of dichotomous EMT marker status for each cut point.

*EMT* epithelial-mesenchymal transition, *NRI* net reclassification index, *ROC* receiver operating characteristic, *TNM* tumor, node, metastasis

## Conclusions

We have illustrated how Bayesian latent class estimation of diagnostic accuracy, and reclassification analysis of prediction increment, are valid and clinically-informative tools to evaluate whether and how an EMT marker should be used clinically to improve tumor staging. This is especially true when continuous marker data are available to be dichotomized at multiple cut points to identify trade-offs between different definitions of high versus low marker expression. Such considerations of cut points, diagnostic accuracy, and prediction increment could allow EMT markers to fulfill their potential to improve diagnosis and treatment decisions for patients with many different kinds of cancer.

## Additional files

Additional file 1: This file has 2 sections providing details about the following: 1) the rationale for the Bayesian priors used for the lymph node evaluation and radiologic imaging diagnostic tests in the latent class analysis of diagnostic accuracy, and 2) sample WinBUGS code for Bayesian latent class estimation of EMT marker sensitivity and specificity. (PDF 779 kb)

Additional file 2: This file provides 4 risk stratification tables (Tables S1-S4) showing classification of subject risk for all-cause mortality based on a model with predictors of lymph node evaluation and radiologic imaging compared to classification based on a model with predictors of lymph node evaluation, radiologic imaging, and E-cadherin measurements in primary tumor cancer cells. Each of the 4 tables is for a different form of the E-cadherin variable (continuous on a scale of 0–3 or dichotomized at 0.52, 0.60, or 0.85). The file also provides several figures: 1) images of high and low E-cadherin immunohistochemistry staining in tumor tissue specimens (Additional file 2: Figure S1), and 2) graphical comparisons of predicted probabilities based only on established diagnostic tests to predicted probabilities based on E-cadherin added to established diagnostic tests (Additional file 2: Figures S2-S5). (PDF 479 kb)

## Abbreviation

CanCORS: Cancer care outcomes research and surveillance consortium
CRC: Colorectal cancer
EMT: Epithelial-mesenchymal transition
IDI: Integrated discrimination improvement
IHC: Immunohistochemistry
NRI: Net reclassification index
ROC: Receiver operating characteristic
TNM: Tumor, node, metastasis
