# Supplementary material for: Diagnostic accuracy and prediction increment of markers of epithelial-mesenchymal transition to assess cancer cell detachment from primary tumors
Source: BMC Cancer. 2018 Jan 16;18:82. doi: 10.1186/s12885-017-3964-3 (PMC5769498; doi:10.1186/s12885-017-3964-3)
Supplement: Supplementary file 1 — This file has 2 sections providing details about the following: 1) the rationale for the Bayesian priors used for the lymph node evaluation and radiologic imaging diagnostic tests in the latent class analysis of diagnostic accuracy, and 2) sample WinBUGS code for Bayesian latent class estimation of EMT marker sensitivity and specificity. (PDF 779 kb) [file 12885_2017_3964_MOESM1_ESM.pdf]

Diagnostic Accuracy and Prediction Increment of Markers of Epithelial-Mesenchymal Transition to Assess Cancer Cell Detachment from Primary Tumors

BMC Cancer

Evan L. Busch, Prabhani Kuruppumullage Don, Haitao Chu, David B. Richardson, Temitope O. Keku, David A. Eberhard, Christy L. Avery, and Robert S. Sandler

Additional File 1: Supplemental Methods

I. Formulation of Bayesian priors for standard clinical tests of cancer cell detachment from primary tumors

The two standard tests of detachment already in clinical use are:

1. Examination of regional lymph nodes (LN)
2. Radiologic imaging, especially of sites distant from the primary tumor (RI)

A diagnosis of local disease (i.e. no detachment of cancer cells from the primary tumor) is only given when both tests are negative.

A false diagnosis of local disease occurs when detachment has taken place but is not detected by either test. In practice, a false diagnosis of local disease requires false negative test results on both standard tests. For simplicity, if we assume that LN and RI results are independent, we get:

p(false diagnosis of local disease) = p(LN false negative result) x p(RI false negative result)

We develop priors for the sensitivity of LN and RI from the above equation and the following background information on colorectal cancer, the kind of cancer studied in our dataset:

a. About 40% of colorectal cancers are diagnosed as being local disease[1]

b. About 25% of colorectal cancer patients diagnosed with local disease (constituting 10% of all colorectal cancer patients) develop recurrent disease after surgery, when in theory this should be impossible or extremely rare. Most of these tumors likely had spread of disease due to cancer cell detachment from the primary that was not detected at diagnosis.[2]

c. Therefore, ~10% of all colorectal cancer diagnoses are false diagnoses of local disease. The other roughly 90% of diagnoses are true or accurate diagnoses of cancer cell detachment (either true finding of detachment or true finding of no detachment).

The above information is summarized in the following diagram:

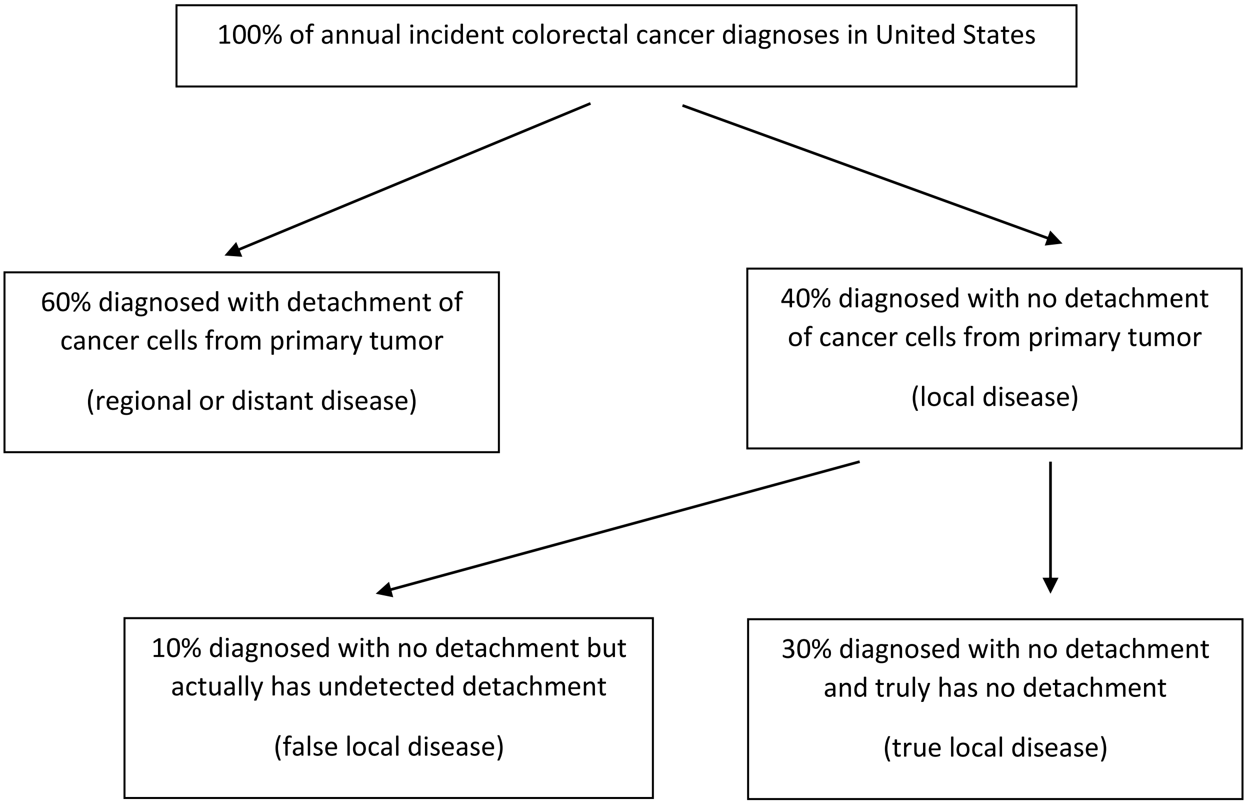

False diagnoses of metastatic disease (i.e. false findings of detachment) are probably rare. For this to happen, a false positive test result would need to occur according to at least one of the two standard tests. This would require a pathologist or radiologist to think he is looking at cancer cells under the microscope or in a scan when in fact he is not. In theory this can happen but should be exceptionally rare.

In the absence of more specific, reliable information for LN or RI, we assume that the probability of a false negative result for LN is the same as the probability of a false negative result for RI. Call this probability Q. The diagram above suggests that about 70% of colorectal cancer patients truly have detachment at diagnosis, and that about 1/7 = ~14.3% of these patients with detachment are falsely diagnosed as not having detachment.

Therefore:

p(false diagnosis of local disease | has detachment) = p(LN false negative) x p(RI false negative)

14.3% = 0.143 = Q x Q = Q^2

Q = square root(0.143) = 0.378 = about 38%

In other words, background information suggests that the "average" false negative percentage for LN and RI is about 38% for each test. Since sensitivity = (100% - false negative percentage), the average sensitivity of the two standard tests is estimated to be (100 – 38)% = 62%. In practice, one of the tests will have a higher sensitivity than this, and the other will have a lower sensitivity, but these are expected to "average out" to about 62%.

Because false positive test results should be very rare for the two standard tests, each test is assumed to have a specificity of close to 100%, with allowance for some error.

The above considerations led to the following prior being applied to each of LN and RI:

Sensitivity: 60%-70%

Specificity: 95%-99%

II. Sample WinBUGS code for Bayesian latent class estimation of EMT marker sensitivity and specificity

Let Y_{ij} be the classification result of the jth of three tests for individual i ($i = 1, \ldots, N$), the latent variable D_i denote the true disease status, and π_i denote the disease probability of the ith subject. The correlation in disease misclassification is accommodated by a latent continuous variable $Z_i \sim N(0, 1)$. The positive result for the jth assessment is assumed to depend on both the latent true disease status D_i of the ith subject and the Gaussian latent variable Z_i through a generalized linear mixed regression model, such as a probit model,[3]

$$P\left(Y_{ij} = 1 \middle| D_i = d_i, Z_i = z_i\right) = \Phi(a_{d_ij} + c_{d_ij} z_i)$$

where d_i = 0,1. Here, the latent Gaussian random variable Z_i is assumed to be independent to the latent disease status D_i . If Se_j and Sp_j denote the sensitivity and specificity for the jth test based on the fully dependent model ($c_{d_ij} \neq 0$), then

$$Se_j = P\left(Y_{ij} = 1 \middle| D_i = 1\right) = \Phi\left(\frac{a_{1j}}{\sqrt{1 + c_{1j}^2}}\right)$$

$$Sp_j = P\left(Y_{ij} = 0 \middle| D_i = 0\right) = 1 - \Phi\left(\frac{a_{0j}}{\sqrt{1 + c_{0j}^2}}\right)$$

Assuming that the multiple exposure assessments are conditionally independent given the latent disease status D_i and latent Gaussian random variable Z_i, the probability of observing $\boldsymbol{Y_i} = (y_{i1}, y_{i2}, y_{i3})$ for the ith subject is

$$P(y_{i1}, y_{i2}, y_{i3}) = \pi_i \int_{-\infty}^{\infty} \prod_{j=1}^{3} \left\{\Phi\left(a_{1j} + c_{1j} z_i\right)^{y_{ij}} \left[1 - \Phi\left(a_{1j} + c_{1j} z_i\right)^{1-y_{ij}}\right]\right\} d\Phi(z_i)$$
$$+ (1 - \pi_i) \int_{-\infty}^{\infty} \prod_{j=1}^{3} \left\{\Phi\left(a_{0j} + c_{0j} z_i\right)^{y_{ij}} \left[1 - \Phi\left(a_{0j} + c_{0j} z_i\right)^{1-y_{ij}}\right]\right\} d\Phi(z_i)$$

The following WINBUGS code estimates the likelihood within the Bayesian framework as per Zhang et al. to estimate $\theta = (a_{11}, a_{12}, a_{13}, a_{01}, a_{02}, a_{03}, c_{11}, c_{12}, c_{13}, c_{01}, c_{02}, c_{03})$.[3] Note that the fully conditionally independent model is obtained by making $c_{d_ij} = 0$ for all tests, and setting $c_{d_ij} = 0$ for some tests produces different partially conditionally dependent models.

```
model{
        C<-0
        for (i in 1:N){
        zero[i]<-0
          for (j in 1:J){
          element1[i,j]<-y[i,j]*log(phi(a1[j]+c1[j]*z[i]))+(1-y[i,j])*log(1-phi(a1[j]+c1[j]*z[i]))
          element2[i,j]<-y[i,j]*log(phi(a0[j]+c0[j]*z[i]))+(1-y[i,j])*log(1-phi(a0[j]+c0[j]*z[i]))
}

          p[i]<-pi.ed[i]*exp(sum(element1[i,1:J]))+(1-pi.ed[i])*exp(sum(element2[i,1:J]))
          pi.ed[i]~dunif(0,1)
          phii[i]<--log(p[i])+C
          zero[i]~dpois(phii[i])
          z[i]~dnorm(0,1)
          }
             for(j in 1:J){
             se[j]<-phi((a1[j])/sqrt(1+pow(c1[j],2)))
             sp[j]<-1-phi((a0[j])/sqrt(1+pow(c0[j],2)))
             c1[j]~dexp(1)
             c0[j]~dexp(1)
             }
                          a1[1]~dunif(0,1)
                          a1[2]~dnorm(0.39,204.08)
                          a1[3]~dnorm(0.39,204.08)
                          a0[1]~dunif(0,1)
                          a0[2]~dnorm(1.99,34.60)
                          a0[3]~dnorm(1.99,34.60)
 }
```

REFERENCES

1. SEER. SEER Stat Fact Sheets: Colon and Rectum Cancer. http://seer.cancer.gov/statfacts/html/colorect.html Accessed 29 August 2016.

2. Young PE, Womeldorph CM, Johnson EK, Maykel JA, Brucher B, Stojadinovic A, Avital I, Nissan A, Steele SR. Early detection of colorectal cancer recurrence in patients undergoing surgery with curative intent: current status and challenges. *Journal of Cancer* 2014;**5**: 262-71.

3. Zhang J, Cole SR, Richardson DB, Chu H. A Bayesian approach to strengthen inference for case-control studies with multiple error-prone exposure assessments. *Statistics in medicine* 2013;**32**: 4426-37.
