# Supplementary material for: Diagnostic accuracy and prediction increment of markers of epithelial-mesenchymal transition to assess cancer cell detachment from primary tumors
Source: BMC Cancer. 2018 Jan 16;18:82. doi: 10.1186/s12885-017-3964-3 (PMC5769498; doi:10.1186/s12885-017-3964-3)
Supplement: Supplementary file 2 — This file provides 4 risk stratification tables (Tables S1-S4) showing classification of subject risk for all-cause mortality based on a model with predictors of lymph node evaluation and radiologic imaging compared to classification based on a model with predictors of lymph node evaluation, radiologic imaging, and E-cadherin measurements in primary tumor cancer cells. Each of the 4 tables is for a different form of the E-cadherin variable (continuous on a scale of 0–3 or dichotomized at 0.52, 0.60, or 0.85). The file also provides several figures: 1) images of high and low E-cadherin immunohistochemistry staining in tumor tissue specimens (Additional file 2: Figure S1), and 2) graphical comparisons of predicted probabilities based only on established diagnostic tests to predicted probabilities based on E-cadherin added to established diagnostic tests (Additional file 2: Figures S2-S5). (PDF 479 kb) [file 12885_2017_3964_MOESM2_ESM.pdf]

Diagnostic Accuracy and Prediction Increment of Markers of Epithelial-Mesenchymal Transition to Assess Cancer Cell Detachment from Primary Tumors

BMC Cancer

Evan L. Busch, Prabhani Kuruppumullage Don, Haitao Chu, David B. Richardson, Temitope O. Keku, David A. Eberhard, Christy L. Avery, and Robert S. Sandler

Additional File 2: Supplemental Results

Table S1. Reclassification of 5-year risk of all-cause mortality among colorectal cancer patients by continuous E-cadherin (n=188)

5-year risk from model without E-cadherin	5-year risk from model with continuous E-cadherin				
	**0-20%**	**20-30%**	**30-40%**	**>40%**	**Total**
**0-20%**					
**Participants, n**	0	0	0	0	0
**Deaths, n**	0	0	0	0	0
**5-year risk, %**	0	0	0	0	0
**20-30%**					
**Participants, n**	40	43	15	1	99
**Deaths, n**	7	10	6	0	23
**5-year risk, %**	17.3	23.0	38.7	0	22.4
**30-40%**					
**Participants, n**	2	12	21	31	66
**Deaths, n**	1	4	4	15	24
**5-year risk, %**	39.3	32.0	18.6	47.6	37.5
**>40%**					
**Participants, n**	0	1	0	22	23
**Deaths, n**	0	0	0	15	15
**5-year risk, %**	0	0	0	66.6	66.0
**Total**					
**Participants, n**	42	56	36	54	188
**Deaths, n**	8	14	10	30	62
**5-year risk, %**	15.4	25.0	34.4	54.8	33.0

Model without E-cadherin includes standard diagnostic tests of lymph node evaluation and radiologic imaging (each coded as dichotomous positive versus negative). For both predictors, a positive result means evidence supporting detachment of cancer cells from the primary tumor and

a negative result means no evidence of detachment. Model with E-cadherin includes standard diagnostic tests and cellular membrane E-cadherin expression measured by immunohistochemistry in primary tumor cancer cells on a continuous average intensity scale (0-3). Both models are Cox proportional hazards models of time from cancer diagnosis to all-cause mortality, censored at 5 years after diagnosis. Cut points to define mortality risk categories were 20%, 30%, and 40%. Numbers of participants and deaths are observed counts. Reported risks are predicted risks.

Table S2. Reclassification of 5-year risk of all-cause mortality among colorectal cancer patients by E-cadherin dichotomized at 0.52 (n=188)

5-year risk from model without E-cadherin	5-year risk from model with E-cadherin dichotomized at 0.52				
	0-20%	20-30%	30-40%	>40%	Total
**0-20%**					
**Participants, n**	0	0	0	0	0
**Deaths, n**	0	0	0	0	0
**5-year risk, %**	0	0	0	0	0
**20-30%**					
**Participants, n**	93	0	0	6	99
**Deaths, n**	20	0	0	3	23
**5-year risk, %**	21.4	0	0	46.0	22.4
**30-40%**					
**Participants, n**	0	0	61	5	66
**Deaths, n**	0	0	20	4	24
**5-year risk, %**	0	0	32.5	72.3	37.5
**>40%**					
**Participants, n**	0	0	0	23	23
**Deaths, n**	0	0	0	15	15
**5-year risk, %**	0	0	0	63.8	66.0
**Total**					
**Participants, n**	93	0	61	34	188
**Deaths, n**	20	0	20	22	62
**5-year risk, %**	20.0	0	34.6	66.4	33.0

Model without E-cadherin includes standard diagnostic tests of lymph node evaluation and radiologic imaging (each coded as dichotomous positive versus negative). Model with E-cadherin includes standard diagnostic tests and cellular membrane E-cadherin expression measured by immunohistochemistry in primary tumor cancer cells on a continuous average intensity scale (0-3), then dichotomized at 0.52 (coded as dichotomous positive versus negative). For all predictors, a positive result means evidence supporting detachment of cancer cells from the primary tumor and a negative result means no evidence of detachment. Both models are Cox proportional hazards models of time from cancer diagnosis to all-cause mortality, censored at 5 years after diagnosis. Cut points to define mortality risk categories were 20%, 30%, and 40%. Numbers of participants and deaths are observed counts. Reported risks are predicted risks.

Table S3. Reclassification of 5-year risk of all-cause mortality among colorectal cancer patients by E-cadherin dichotomized at 0.60 (n=188)

5-year risk from model without E-cadherin	5-year risk from model with E-cadherin dichotomized at 0.60				
	0-20%	20-30%	30-40%	>40%	Total
**0-20%**					
Participants, n	0	0	0	0	0
Deaths, n	0	0	0	0	0
5-year risk, %	0	0	0	0	0
**20-30%**					
Participants, n	83	0	0	16	99
Deaths, n	17	0	0	6	23
5-year risk, %	20.3	0	0	36.3	22.4
**30-40%**					
Participants, n	0	0	55	11	66
Deaths, n	0	0	17	7	24
5-year risk, %	0	0	30.6	60.8	37.5
**>40%**					
Participants, n	0	0	0	23	23
Deaths, n	0	0	0	15	15
5-year risk, %	0	0	0	63.8	66.0
**Total**					
Participants, n	83	0	55	50	188
Deaths, n	17	0	17	28	62
5-year risk, %	19.0	0	33.0	56.7	33.0

Model without E-cadherin includes standard diagnostic tests of lymph node evaluation and radiologic imaging (each coded as dichotomous positive versus negative). Model with E-cadherin includes standard diagnostic tests and cellular membrane E-cadherin expression measured by immunohistochemistry in primary tumor cancer cells on a continuous average intensity scale (0-3), then dichotomized at 0.60 (coded as dichotomous positive versus negative). For all predictors, a positive result means evidence supporting detachment of cancer cells from the primary tumor and a negative result means no evidence of detachment. Both models are Cox proportional hazards models of time from cancer diagnosis to all-cause mortality, censored at 5 years after diagnosis. Cut points to define mortality risk categories were 20%, 30%, and 40%. Numbers of participants and deaths are observed counts. Reported risks are predicted risks.

Table S4. Reclassification of 5-year risk of all-cause mortality among colorectal cancer patients by E-cadherin dichotomized at 0.85 (n=188)

**5-year risk from model without E-cadherin**	**5-year risk from model with E-cadherin dichotomized at 0.85**				
	**0-20%**	**20-30%**	**30-40%**	**>40%**	**Total**
**0-20%**					
**Participants, n**	0	0	0	0	0
**Deaths, n**	0	0	0	0	0
**5-year risk, %**	0	0	0	0	0
**20-30%**					
**Participants, n**	43	56	0	0	99
**Deaths, n**	8	15	0	0	23
**5-year risk, %**	18.3	26.5	0	0	22.4
**30-40%**					
**Participants, n**	0	25	0	41	66
**Deaths, n**	0	7	0	17	24
**5-year risk, %**	0	27.4	0	41.0	37.5
**>40%**					
**Participants, n**	0	0	2	21	23
**Deaths, n**	0	0	0	15	15
**5-year risk, %**	0	0	0	69.7	66.0
**Total**					
**Participants, n**	43	81	2	62	188
**Deaths, n**	8	22	0	32	62
**5-year risk, %**	15.0	27.3	38.0	53.3	33.0

Model without E-cadherin includes standard diagnostic tests of lymph node evaluation and radiologic imaging (each coded as dichotomous positive versus negative). Model with E-cadherin includes standard diagnostic tests and cellular membrane E-cadherin expression measured by immunohistochemistry in primary tumor cancer cells on a continuous average intensity scale (0-3), then dichotomized at 0.85 (coded as dichotomous positive versus negative). For all predictors, a positive result means evidence supporting detachment of cancer cells from the primary tumor and a negative result means no evidence of detachment. Both models are Cox proportional hazards models of time from cancer diagnosis to all-cause mortality, censored at 5 years after diagnosis. Cut points to define mortality risk categories were 20%, 30%, and 40%. Numbers of participants and deaths are observed counts. Reported risks are predicted risks.

<u>Figure S1.</u> <u>E-cadherin immunohistochemistry staining of positive and negative cores</u>

A. Positive staining

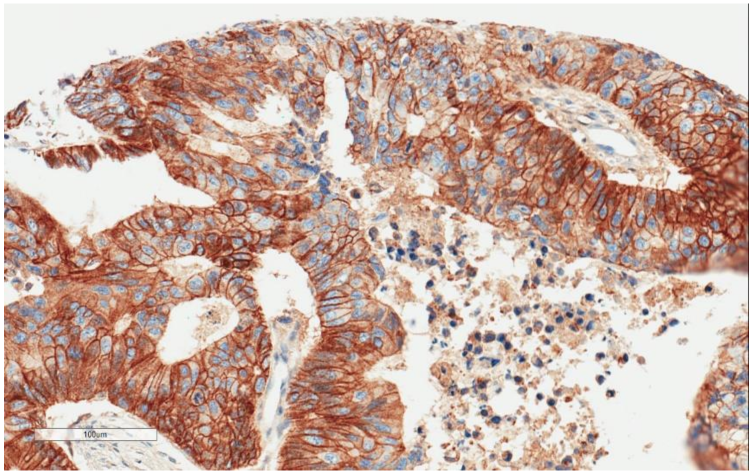

B. Negative staining

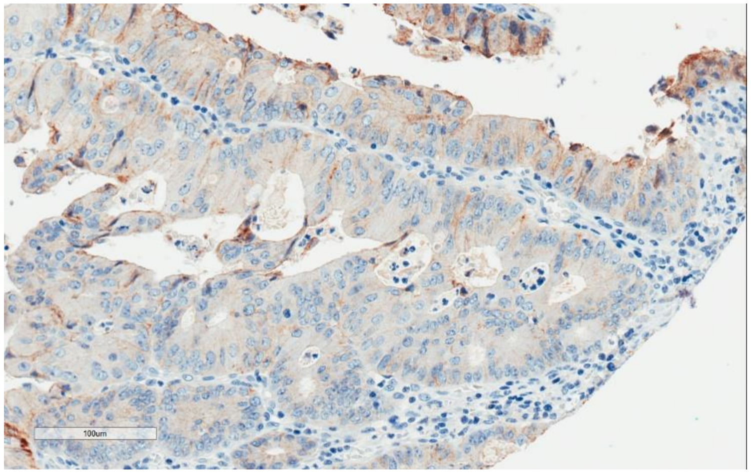

Reproduced from Springer *Clinical & Experimental Metastasis*, Evaluating markers of epithelial-mesenchymal transition to identify cancer patients at risk for metastatic disease, Volume 33 (1), 2016, Supplementary material 3 (page 4, panels A and B), Figure S3: Immunohistochemistry examples for positive and negative core staining for E-cadherin and Snail [A) E-cadherin positive, B) E-cadherin negative] by Evan L. Busch, Temitope O. Keku, David B. Richardson, Stephanie M. Cohen, David A. Eberhard, Christy L. Avery, and Robert S. Sandler. Copyright Springer Science+Business Media Dordrecht 2015. With permission of Springer.

Figure S2. Predicted probabilities of colorectal cancer case all-cause mortality within 5 years of diagnosis for predictors of lymph node evaluation and radiologic imaging, either with or without further predictor of continuous E-cadherin (n=188)

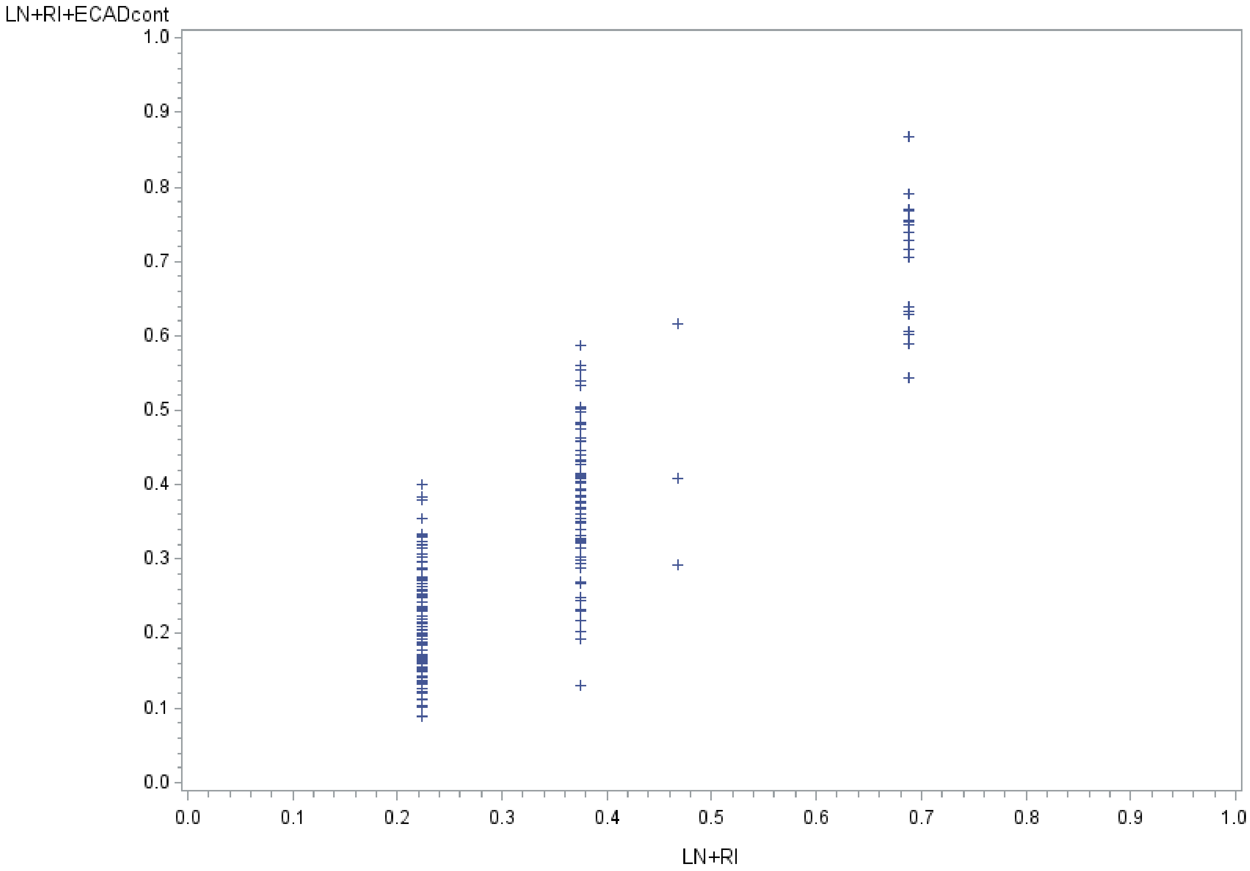

All models were Cox proportional hazards models of time from colorectal cancer diagnosis to all-cause mortality, censored at 5 years. Models included predictors of lymph node evaluation (present vs. absent cancer cells) and radiologic imaging (present vs. absent cancer cells), and either did or did not further include measurement of E-cadherin in primary tumor cancer cells (continuous average intensity scale of 0-3).

LN=lymph node evaluation, ECADcont=continuous E-cadherin, RI=radiologic imaging

Figure S3. Predicted probabilities of colorectal cancer case all-cause mortality within 5 years of diagnosis for predictors of lymph node evaluation and radiologic imaging, either with or without further predictor of E-cadherin dichotomized at 0.52 (n=188)

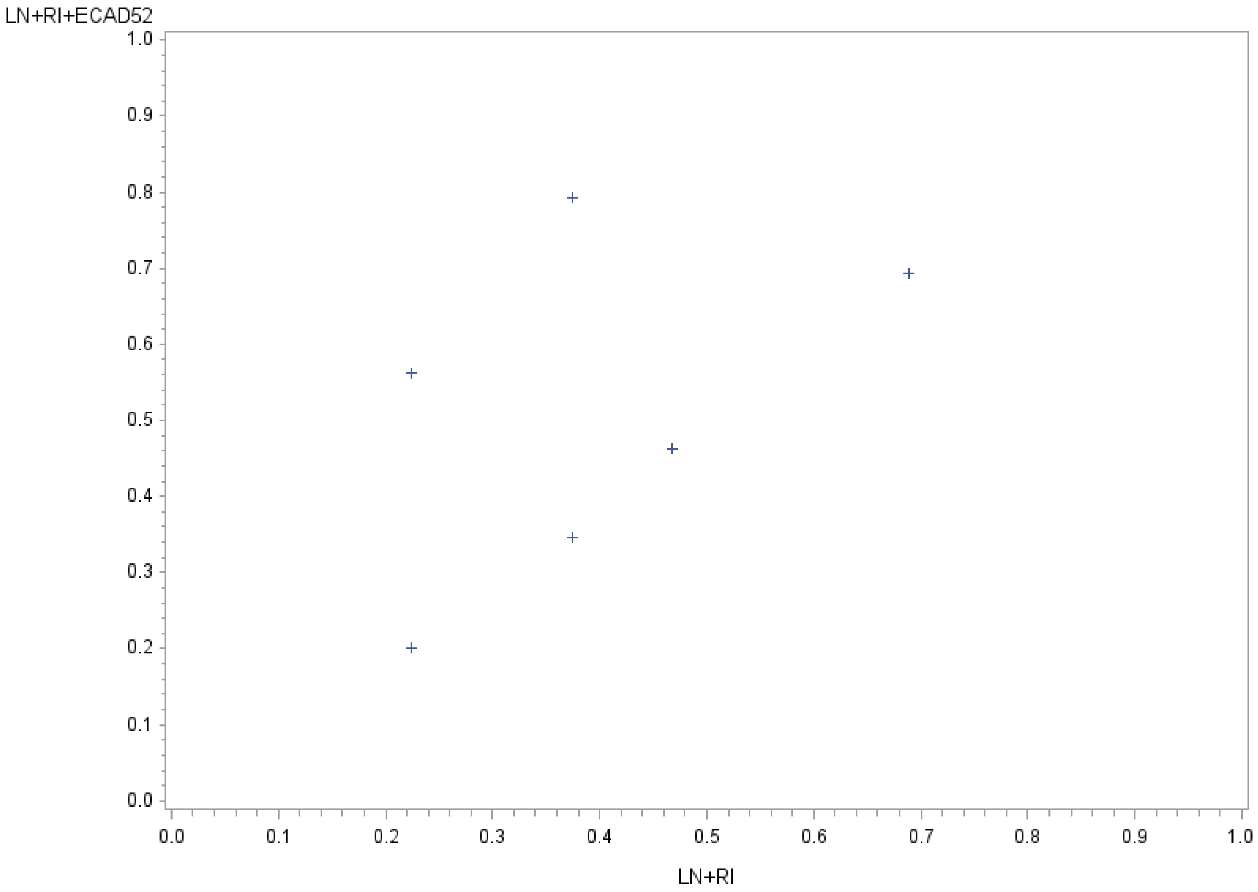

All models were Cox proportional hazards models of time from colorectal cancer diagnosis to all-cause mortality, censored at 5 years. Models included predictors of lymph node evaluation (present vs. absent cancer cells) and radiologic imaging (present vs. absent cancer cells), and either did or did not further include measurement of E-cadherin in primary tumor cancer cells (continuous average intensity scale of 0-3, then dichotomized at 0.52).

LN=lymph node evaluation, ECAD52=E-cadherin dichotomized at 0.52, RI=radiologic imaging

Figure S4. Predicted probabilities of colorectal cancer case all-cause mortality within 5 years of diagnosis for predictors of lymph node evaluation and radiologic imaging, either with or without further predictor of E-cadherin dichotomized at 0.60 (n=188)

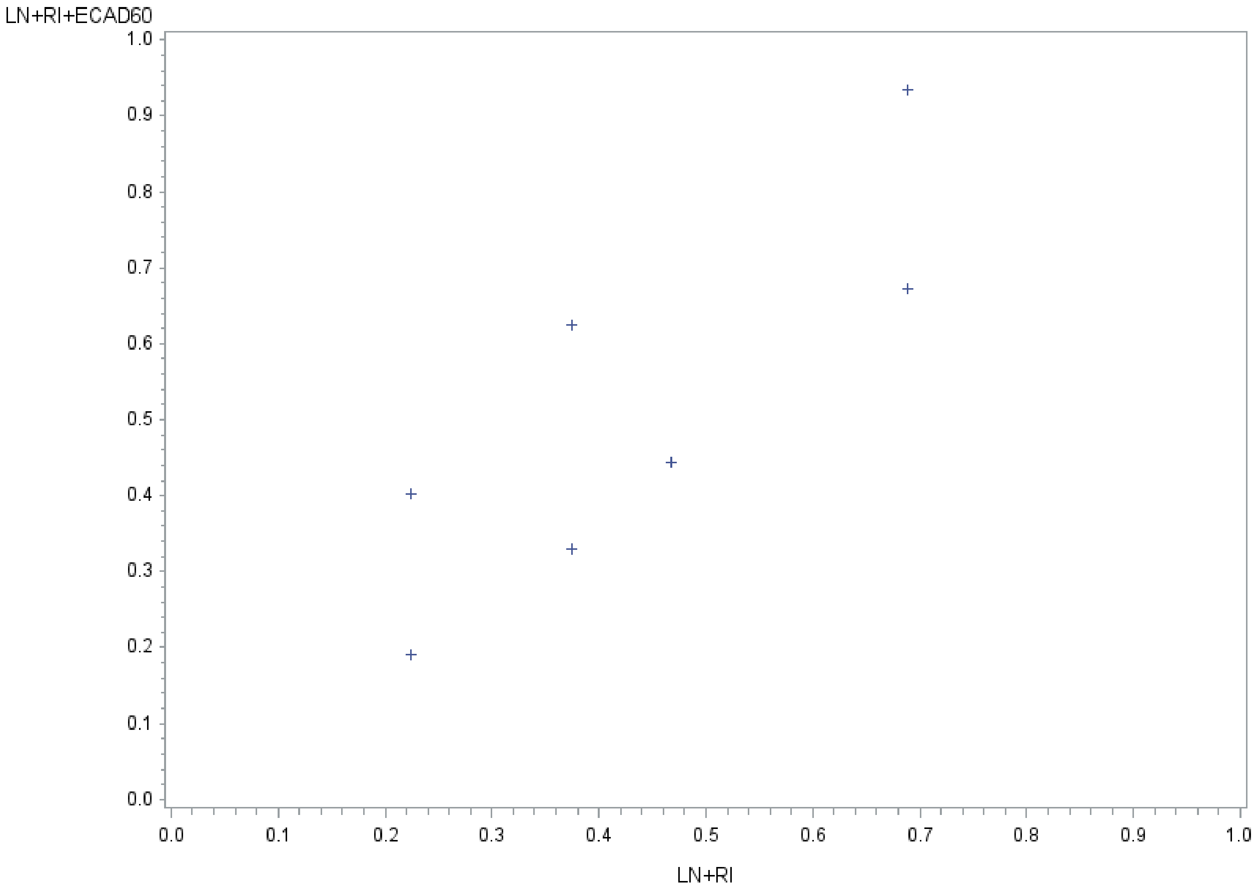

All models were Cox proportional hazards models of time from colorectal cancer diagnosis to all-cause mortality, censored at 5 years. Models included predictors of lymph node evaluation (present vs. absent cancer cells) and radiologic imaging (present vs. absent cancer cells), and either did or did not further include measurement of E-cadherin in primary tumor cancer cells (continuous average intensity scale of 0-3, then dichotomized at 0.60).

LN=lymph node evaluation, ECAD60=E-cadherin dichotomized at 0.60, RI=radiologic imaging

Figure S5. Predicted probabilities of colorectal cancer case all-cause mortality within 5 years of diagnosis for predictors of lymph node evaluation and radiologic imaging, either with or without further predictor of E-cadherin dichotomized at 0.85 (n=188)

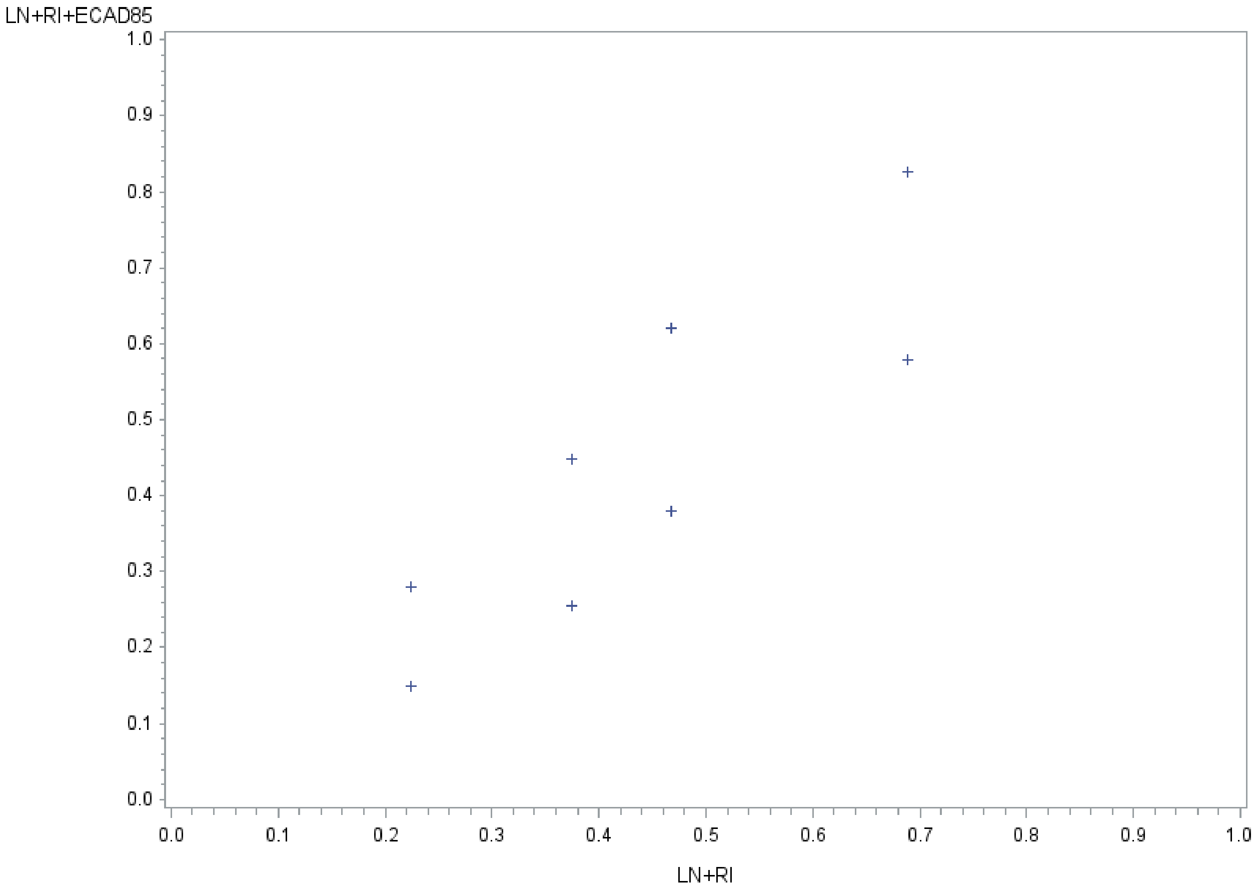

All models were Cox proportional hazards models of time from colorectal cancer diagnosis to all-cause mortality, censored at 5 years. Models included predictors of lymph node evaluation (present vs. absent cancer cells) and radiologic imaging (present vs. absent cancer cells), and either did or did not further include measurement of E-cadherin in primary tumor cancer cells (continuous average intensity scale of 0-3, then dichotomized at 0.85).

LN=lymph node evaluation, ECAD85=E-cadherin dichotomized at 0.85, RI=radiologic imaging
